# The Story of the Insane from Year to Year

**Published:** 1896-10-10

**Authors:** 


### THE STORY OF THE INSANE FROM YEAR TO YEAR.

Ninth Series.

### OXFORD COUNTY ASYLUM AT LITTLEMORE.

During the year 1895 the numbers increased from 523 to 535. There were 140 admissions, 34 recoveries, 12 discharged relieved, and 73 deaths. The recoveries, calculated on the admissions, stand at 24 per cent. and the deaths at 14 per cent. on the average number resident; the former is below the County Asylum average and the latter above it. Of the deaths 17 were from brain diseases and 21 from lung diseases. Of the causes of insanity in the year's admissions, we find drink heading the list with 19; heredity follows with 15, while various bodily diseases have 24 cases assigned to them. Dr. Good, the assistant medical officer, gives lectures on mental nursing to the staff, and ten of the attendants have obtained their nursing certificates. Dr. Sankey had one very unpleasant experience during the year. The female side of the building caught fire, and could not be put out until the roof and second floor of a wing 200 feet long had been burned. Temporary arrangements were at once made to cope with the loss of space entailed by the destruction, and much energy was shown in putting everything right again, for in ten weeks the work was finished. The Committee of Visitors traverse the Commissioners' report, and "venture to deny the accuracy of the statement that 'the entire building was gutted' by the fire." The Commissioners say "the asylum appliances in a measure broke down," while the Committee reply that "the asylum fire appliances were in good working order throughout the day." The Commissioners say "there still exist certain places which have no alternative exits," and the "Committee are satisfied that alternative exits are provided for all the wards in case of fire." Who can decide when doctors disagree? The weekly charge to the Unions is 7s. 6d. per head, and the actual cost a little less than this sum.

### DERBY BOROUGH ASYLUM.

Here the numbers were almost stationary during the year 1895, being 310 on January 1st and 311 on the last day of December. There were 96 admissions, 38 recoveries, 10 discharged relieved, and 38 deaths. The recoveries stand at the very creditable percentage of 46·3 on the admissions, and the deaths are a little above the average at 12·2 on the average number resident. Seventeen of the deaths were caused by cerebral and spinal diseases and four by consumption. Turning to table 10, which shows the causes of insanity, in the year's admissions we find that 25 cases were owing to drink and 36 to hereditary influence; that is, 61 out of a total of 96 were from preventible causes and ought not to have existed at all. This journal has often brought these facts under public notice during the last nine years; and although no result may be perceptible we do not mean to give up hope of ultimately doing good. Five coroners' inquests were held during the year, a group which Dr. Macphail will, no doubt, think should confer immunity from such visitations for many years to come. As might have been expected in an asylum superintended by Dr. Macphail, no blame was in any case attributed by the jury to anyone in the asylum. The medical officers continue their lectures and demonstrations to the nursing staff, and it is to be hoped the latter will not stop at the medico-psychological certificate, but will proceed much further with their studies. We note with much satisfaction that several of the nurses have joined the Royal National Pension Fund. The committee say they have "entire satisfaction with the manner in which Dr. Macphail continues to discharge the onerous and responsible duties which devolve upon him." We did not notice the usual table showing the average weekly cost per head, but in the previous year it was 8s. 6½d.

### HOLLOWAY SANATORIUM, VIRGINIA WATER.

This hospital contained 350 patients on January 1st, 1895, and 328 on the last day of the year. There were 122 admissions, 72 recoveries, 36 discharged relieved, and 13 deaths. The recoveries stand at the high percentage of 64·86 on the admissions, and the deaths are only 3·81 on the average number resident. Drink figures as the cause of insanity in 11 of the admissions, hereditary influence in 18, domestic trouble in 6, and religion in 12. Seven of the deaths were owing to cerebral diseases and 1 to consumption. All sorts and conditions of men were admitted during the year. We notice clergymen, officers in the army and navy, engineers, physicians, novelists, schoolmasters, bankers, &c. The hospital seems to be in an efficient state—the recovery rate was high, the death-rate low, and there was only one accident, and that a minor one. There was no epidemic disease and no suicide. Regarding suicides in asylums, Dr. Philipps truly says that "no human precautions will make suicide absolutely unknown in any asylum, and the very precautions taken in many cases to prevent suicide often ruin the prospects of recovery." The entertainments given to the patients were very numerous. During the past year 109 took place, and in addition to these 143 patients were sent to Brighton for part of the year. When referring to the well-known Weir case, Dr. Philipps says "the *Truth* attacks have cost the committee some time and trouble, but not a single patient has been removed from the sanatorium in consequence of them." This is one of the few English asylums which has a "lady medical" on the staff, and we are pleased to learn that a female medical officer is considered a valuable auxiliary at Virginia Water. The committee say they believe "the hospital staff is complete and satisfactory in every way, and that notwithstanding the attacks that have been made upon it, the demand for accommodation on the part of patients of the best class continues satisfactory, and the balance at credit shows a large increase." The total amount given in charity is stated at £7,760.

BELFAST DISTRICT ASYLUM.

The limits of accommodation are stated to be 400; but there were 750 in residence at the beginning of the year, and at the end of 1895 this number had risen to 810. There were 240 admissions, 90 recoveries, 40 discharged relieved, and 44 deaths. The percentage of recoveries on admissions is 37·5, and that of the deaths on the average number resident is 5·6. Of the deaths, 26 were caused by cerebral and spinal diseases, and 6 by consumption. Drink is the assigned cause of insanity in 22 of the year's admissions, hereditary influence in 25, uterine disorders in 21, religion in 15, and domestic trouble in 26. Dr. Merrick complains of the difficulty of proper treatment and classification caused by the overcrowding, and we can readily believe that he has his hands full. In spite of the overcrowding, the health of the patients must be good, for the death-rate is low. The cost per head per annum is £22 1s. 6d.

